# Oral Tolerance to Environmental Mycobacteria Interferes with Intradermal, but Not Pulmonary, Immunization against Tuberculosis

**DOI:** 10.1371/journal.ppat.1005614

**Published:** 2016-05-06

**Authors:** Dominique N. Price, Donna F. Kusewitt, Christopher A. Lino, Amber A. McBride, Pavan Muttil

**Affiliations:** 1 Department of Pharmaceutical Sciences, College of Pharmacy, University of New Mexico, Albuquerque, New Mexico, United States of America; 2 Biomedical Sciences Graduate Program, University of New Mexico, Albuquerque, New Mexico, United States of America; 3 Department of Pathology, University of New Mexico, Albuquerque, New Mexico, United States of America; 4 Department of Molecular Genetics and Microbiology, University of New Mexico, Albuquerque, New Mexico, United States of America; 5 Sandia National Laboratories, Albuquerque, New Mexico, United States of America; McGill University Health Centre, CANADA

## Abstract

Bacille Calmette–Guérin (BCG) is currently the only approved vaccine against tuberculosis (TB) and is administered in over 150 countries worldwide. Despite its widespread use, the vaccine has a variable protective efficacy of 0–80%, with the lowest efficacy rates in tropical regions where TB is most prevalent. This variability is partially due to ubiquitous environmental mycobacteria (EM) found in soil and water sources, with high EM prevalence coinciding with areas of poor vaccine efficacy. In an effort to elucidate the mechanisms underlying EM interference with BCG vaccine efficacy, we exposed mice chronically to *Mycobacterium avium (M*. *avium)*, a specific EM, by two different routes, the oral and intradermal route, to mimic human exposure. After intradermal BCG immunization in mice exposed to oral *M*. *avium*, we saw a significant decrease in the pro-inflammatory cytokine IFN-γ, and an increase in T regulatory cells and the immunosuppressive cytokine IL-10 compared to naïve BCG-vaccinated animals. To circumvent the immunosuppressive effect of oral *M*. *avium* exposure, we vaccinated mice by the pulmonary route with BCG. Inhaled BCG immunization rescued IFN-γ levels and increased CD4 and CD8 T cell recruitment into airways in *M*. *avium*-presensitized mice. In contrast, intradermal BCG vaccination was ineffective at T cell recruitment into the airway. Pulmonary BCG vaccination proved protective against Mtb infection regardless of previous oral *M*. *avium* exposure, compared to intradermal BCG immunization. In conclusion, our data indicate that vaccination against TB by the pulmonary route increases BCG vaccine efficacy by avoiding the immunosuppressive interference generated by chronic oral exposure to EM. This has implications in TB-burdened countries where drug resistance is on the rise and health care options are limited due to economic considerations. A successful vaccine against TB is necessary in these areas as it is both effective and economical.

## Author Summary

The current vaccine against tuberculosis (TB), BCG, has variable efficacy (0–80%) at protecting against infection. A large body of clinical and experimental evidence implicates host exposure to environmental mycobacteria (EM) as the cause of interference with BCG vaccine-induced immunity. We explore the mechanism by which EM interferes with parenteral immunization and propose oral tolerance as a mechanism for this interference. In addition, we use the pulmonary route to vaccinate EM-exposed hosts, thus ensuring effective BCG immunization and subsequent protection against TB. This work has broad implications in the TB vaccine field as it shows that the vulnerability of the current vaccine, and that of many novel vaccine candidates, may be the route of administration and not necessarily the vaccine itself.

## Introduction

Tuberculosis (TB) is one of the leading causes of death due to an infectious agent worldwide, with a yearly mortality of 1.5 million [1,2]. The World Health Organization estimates that over one-third of the global population is latently infected with *Mycobacterium tuberculosis* (Mtb), the bacterium that causes TB, and every year 9 million people develop active TB. These mortality and morbidity statistics, coupled with increasingly limited antibiotic options due to drug resistance, demonstrate why an effective vaccine against TB has become a global health priority [1,2].

Bacille Calmette-Guérin (BCG) is the only approved vaccine against TB. Despite widespread use, BCG offers variable protection against pulmonary disease (0–80%), with the lowest efficacy in geographical regions where TB is most prevalent [3]. This variability is due, at least in part, to environmental mycobacteria (EM) exposure [3–9]. EM are present ubiquitously in soil and water sources around the world, with high concentrations coinciding with areas of poor vaccine efficacy [10–12]. Individuals living in these areas have high EM exposure via the gastrointestinal (GI) tract [12]. While EM interference with BCG protection has been widely studied, the mechanism of action has not been elucidated and continues to be a topic of debate [3,6,13–15].

To elucidate the mechanism of interference by EM, it is necessary to understand how the immune system responds to EM and BCG, separately and together, in the appropriate tissue of exposure. Homeostasis, inflammation, and tolerance are all critical for protection against, and recovery from, pathogens [16–19]. This regulation of immunity is different depending on the location of infection within the body. Localized infections are usually compartmentalized to a specific tissue or niche within the body that favors pathogenesis. Similarly, various tissues within the body can be immunologically primed to home immune cells in a tissue-specific manner, such that these cells manifest their effects primarily in a particular tissue compartment [16,20,21]. Thus, the route of exposure to an antigen predisposes an individual for all subsequent immune responses to the same antigen.

Compartmentalized immunity is an evolutionary advantage. For example, the GI tract needs to respond to pathogens differently than the respiratory tract. The GI tract comes into contact with many pathogens daily and requires mechanisms for generating a tolerogenic response to commonly ingested antigens and pathogens, in order to prevent unnecessary inflammation [22–25]. This phenomena, called oral tolerance, is defined as the lack of a systemic immune response to parenteral immunization with an antigen to which an animal has previously been exposed to through the GI tract [24]. Conversely, the lung and airway are vulnerable to serious infection. The airway is a highly compartmentalized and reactive tissue. This was illustrated by Xing and colleagues, who demonstrated the failures of systemic vaccination against pulmonary pathogens like Mtb [26–28]. They showed that parenteral vaccination with BCG was not sufficient for the recruitment of antigen-specific immune cells to the airway of the lung in a mouse model of TB. Only mucosal (intranasal) immunization generated immune cells in the airway and was protective against subsequent aerosol Mtb infection [28,29].

In the present study, we explored the oral and parenteral routes of EM exposure and their ability to interfere with intradermal BCG immunization. We hypothesized that chronic EM exposure by the oral route results in systemic tolerance toward these EM. We found that this tolerance is cross-reactive to the intradermally delivered BCG vaccine which has cell wall components markedly similar to EM. Consequently, the host does not generate immunity against BCG, and individuals remain vulnerable to TB infection. Furthermore, we found that targeting the immunologically naïve airway by pulmonary immunization circumvented systemic EM immunosuppression and provided BCG-specific immunity which was protective against TB. Our research helps define the mechanism of EM interference with BCG vaccination and indicates that pulmonary administration enhances the protective effects of the BCG vaccine, regardless of prior EM exposure. It is likely that these findings will also be pertinent to future TB vaccines containing mycobacterial antigens that are administered by the parenteral route.

## Results

### Murine T cells are cross-reactive to EM and BCG

**Fig 1:**
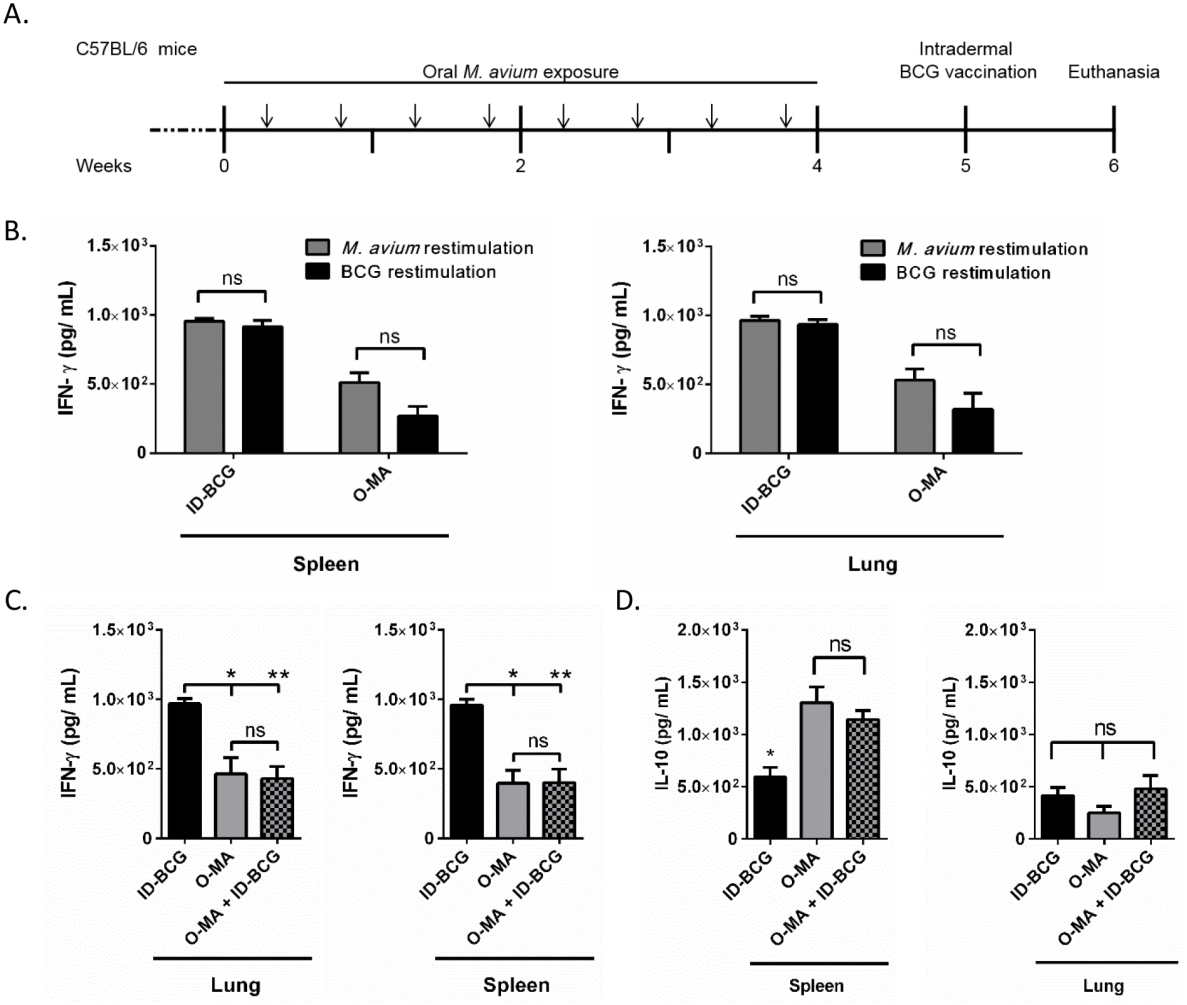
Differences in cytokine response to BCG immunization were observed between mice that were or were not presensitized to oral *M*. *avium* prior to intradermal BCG vaccination. (A) Experimental design. Arrows indicate *M*. *avium* exposure. (B) Interferon-gamma (IFN-γ) secretion in response to restimulation of splenic and lung T cells from mice exposed to either intradermal BCG or oral *M*. *avium* with either heat-killed whole BCG or *M*. *avium*, n = 6. (C) IFN-γ secretion in response to heat-killed whole BCG antigen restimulation of splenic and lung T cells from different treatment groups, n = 6. (D) Interleukin-10 (IL-10) secretion in response to BCG restimulation of splenic and lung T cells from groups intradermally immunized with BCG with and without oral *M*. *avium* presensitization, n = 3. A Mann-Whitney U test or multiple t-tests (Holm-Sidak) were used for comparison of two data sets, and a one-way ANOVA Kruskal-Wallis with Dunn’s multiple comparison post-test for multiple data sets. *p < .05; **p < .01; data shown with standard error of the mean (SEM). Abbreviations: Intradermal BCG only (ID-BCG), oral *M*. *avium* only (O-MA), oral *M*. *avium* presensitization with intradermal BCG vaccination (O-MA + ID-BCG).

To validate EM suppression of parenteral BCG immunization responses, we exposed C57BL/6 mice to *M*. *avium* (a species of EM) by oral gavage a total of eight times over a four-week period (Fig 1A). The exposure regimen was designed to mimic chronic human exposure to EM by ingestion. Mice were rested for one week after the last *M*. *avium* exposure and then immunized with BCG by the intradermal route, which is the current immunization route in humans. Treatment groups included i) oral *M*. *avium* exposure and no subsequent BCG immunization (O-MA), ii) intradermal BCG immunization without prior *M*. *avium* exposure (ID-BCG) or iii) oral *M*. *avium* exposure followed by intradermal BCG immunization (O-MA + ID-BCG). Mice were sacrificed one week after BCG vaccination (6 weeks after initial *M*. *avium* exposure). Mouse spleens and lungs were then harvested and T cells from these tissues were assessed for IFN-γ production after restimulation with heat-killed BCG or *M*. *avium* to determine early responses after vaccination.

T cells were first assessed for antigen cross-reactivity to determine whether lymphocytes from EM-presensitized mice can distinguish between EM and BCG and thus respond differentially. Splenic and lung T cells from O-MA and ID-BCG mice were restimulated with either heat-killed *M*. *avium* or BCG. There was no difference in IFN-γ response with respect to the antigen (*M*. *avium* or BCG) used for restimulation in either tissue (Fig 1B), suggesting marked antigenic similarity between *M*. *avium* and BCG. However, T cells from ID-BCG animals responded with higher IFN-γ secretion than T cells from O-MA animals. Specifically, when restimulated with BCG, splenic T cells from ID-BCG mice secreted high levels of IFN-γ (1x10^3^ pg/mL), whereas T cells from O-MA mice secreted two-fold less (Fig 1C). Importantly, mice presensitized with oral *M*. *avium* before intradermal immunization with BCG (O-MA + ID-BCG) had markedly reduced IFN-γ secretion compared to the ID-BCG group. Indeed, pretreatment with oral *M*. *avium* reduced IFN-γ secretion by T cells from BCG-immunized mice restimulated with BCG to the level seen in mice never immunized with BCG (O-MA only). Likewise, BCG stimulated lung T cells from mice orally exposed to *M*. *avium* prior to intradermal BCG immunization (O-MA + ID-BCG) secreted IFN-γ at levels similar to that of mice treated with oral *M*. *avium* alone (Fig 1C). These data confirm previous findings and show that while intradermal BCG induces a strong proinflammatory response in mice, the proinflammatory response to oral *M*. *avium* exposure is limited [3,9]. Moreover, mice presensitized to oral *M*. *avium* and immunized with BCG responded like unimmunized mice receiving only oral *M*. *avium*. Thus, oral exposure to *M*. *avium* markedly suppressed the IFN-γ response to BCG restimulation.

### Increased tolergenic immune profiles associated with oral EM exposure

Decreased IFN-γ in mice exposed to *M*. *avium* suggested that O-MA suppresses the Th1 response to the BCG vaccine. To further assess the Th1-suppressive effect of EM oral exposure on intradermal BCG immunization in mice, we also assessed differences in T cell production of the pro-inflammatory cytokine TNF-α and the anti-inflammatory cytokine IL-10 following restimulation with BCG. Interestingly, the only significant difference between ID-BCG and O-MA+ID-BCG mice was seen for IL-10. TNF-α was not detectable in any of the groups. Oral *M*. *avium* exposure increased IL-10 levels in the spleen by nearly 2-fold with and without BCG vaccination, compared to BCG alone (Fig 1D). IL-10 is an anti-inflammatory cytokine that is mainly secreted by regulatory T cells (Treg), which are critical for lymphocyte suppression and known mediators of oral tolerance. Furthermore, IL-10 is often increased in tolerogenic systems [22]. Therefore, this data suggested that a tolerance mechanism is involved in the immunosuppressive effects of oral *M*. *avium* in response to subsequent intradermal BCG vaccination.

**Fig 2:**
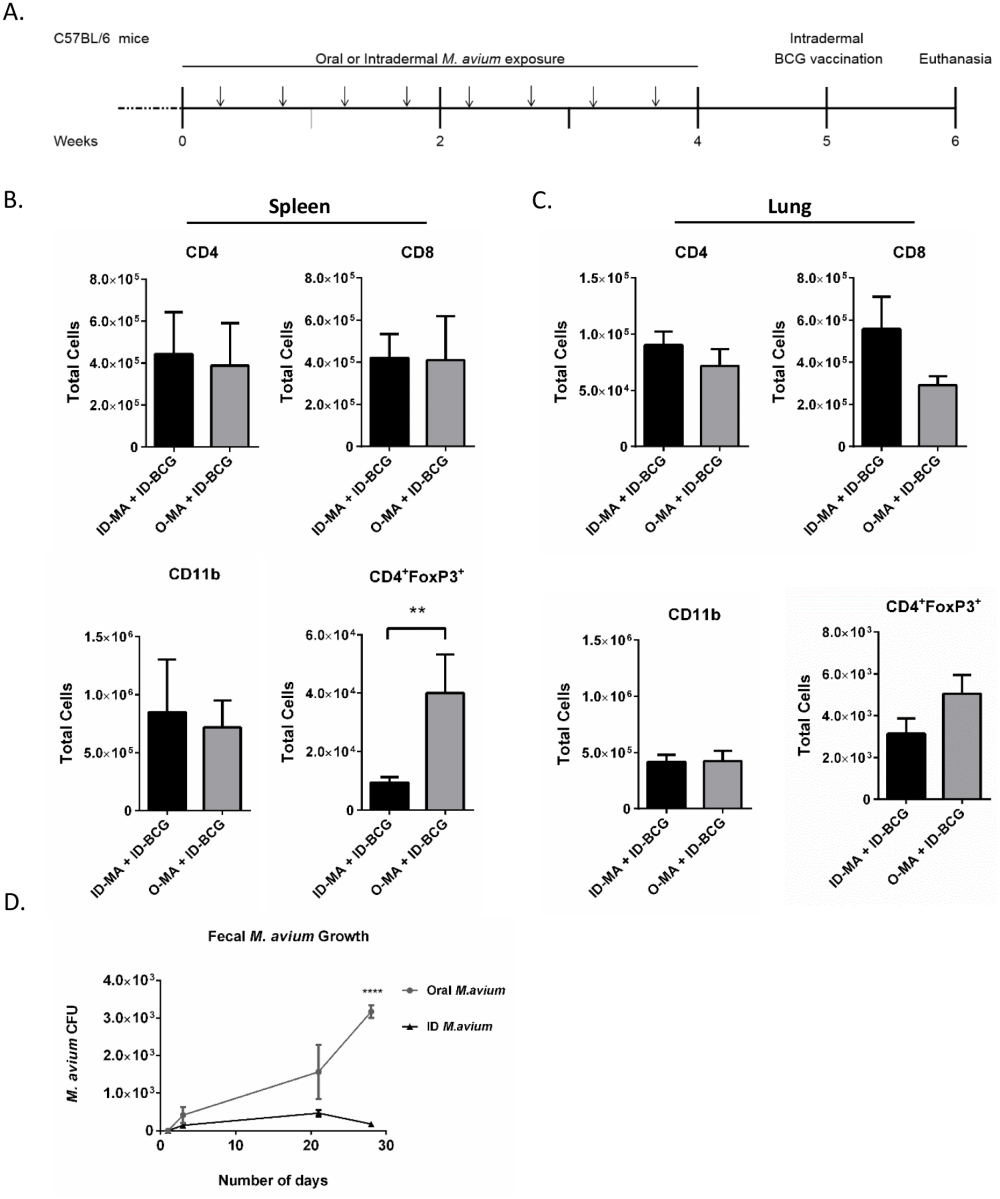
Analysis of cell phenotypes present in spleen and lung tissues after oral or intradermal presensitization to *M*. *avium*. (A) Experimental design. Arrows indicate *M*. *avium* exposure. (B & C) Number of CD4^+^, CD8^+^, CD11b^+^, and CD4^+^FoxP3^+^ cells in the spleens (B) and lungs (C) of mice; n = 8 over two separate experiments. (D) Number of *M*. *avium* CFU in the pooled feces of mice presensitized to *M*. *avium* by different exposure routes. Data collected over two separate experiments (n = 8). A Mann-Whitney U test was used for comparison.*p < .05; **p < .01; ***p < .001 data shown with standard error of the mean (SEM). Abbreviations: Intradermal *M*. *avium* presensitization with intradermal BCG vaccination (ID-MA + ID-BCG), oral *M*. *avium* presensitization with intradermal BCG vaccination (O-MA + ID-BCG).

To further examine the mechanism of EM immunosuppression of BCG immunization, mice were chronically exposed to EM by two different exposure routes: oral (O-MA) and intradermal (ID-MA). The same exposure and dosing regimen used earlier was employed (Fig 2A), with an additional group which received intradermal *M*. *avium* exposure, followed by ID-BCG. Immune cells were assessed 6 weeks after initial *M*. *avium* exposure. Mice exposed to O-MA versus ID-MA prior to ID-BCG had similar splenic T cell (CD4^+^ and CD8^+^) and macrophage (CD11b) numbers. However, O-MA mice had 4-fold more splenic Treg (CD4^+^FoxP3^+^) cells (Fig 2B). Lung tissue showed similar trends, with Treg levels 2-fold higher in the O-MA group. In addition, pulmonary CD8^+^ cells were lower in the O-MA group; however, this difference was not significant (Fig 2C). Cytokine analysis of the tissues showed significantly decreased IFN-γ levels in spleens of O-MA versus ID-MA mice (S1B Fig) and no detectable IFN-γ in the lung. While differences in IL-10 secretion were not significant in either tissue, ID-MA treatment resulted in less IL-10 in the lung than O-MA (S1C Fig). These data suggest that oral *M*. *avium* exposure generates a tolerogenic phenotype in mice and causes the immunosuppressive effects observed in BCG immunized mice.

### Mycobacterial colonization is dependent on route of exposure

We hypothesized if the immune system is not clearing EM from the GI tract due to an oral tolerance mechanism, then *M*. *avium* should be present in the feces of the orally-exposed mice [30,31]. Therefore, it was important to demonstrate bacterial colonization and survival in the gut as an outcome of this oral tolerance model. To assess this, feces from each group were pooled and plated for CFU determinations. No *M*. *avium* was detected in feces from either treatment group on day 1 after the initial exposure (Fig 2D). However, feces from O-MA mice taken on the last day of chronic *M*. *avium* exposure (Day 21), showed higher *M*. *avium* CFUs (1.5 x10^3^), compared to 3-fold fewer CFUs (4.7 x10^2^) in the feces of ID-MA mice (Fig 2D). Feces collected one week after the last *M*. *avium* exposure and just prior to BCG immunization (Day 28) showed that orally administered *M*. *avium* persists one week after the last exposure, suggesting GI tract colonization by *M*. *avium*. While this data should be interpreted with caution because it represents pooled fecal samples, it further supports the hypothesis that chronic *M*. *avium* exposure establishes oral tolerance to mycobacteria in the GI tract of hosts. Systemic *M*. *avium* numbers were also assessed after sacrifice in both the spleens and lungs of mice, and the O-MA group had higher CFUs in both tissues (S2 Fig).

### Pulmonary versus intradermal BCG immunity

**Fig 3:**
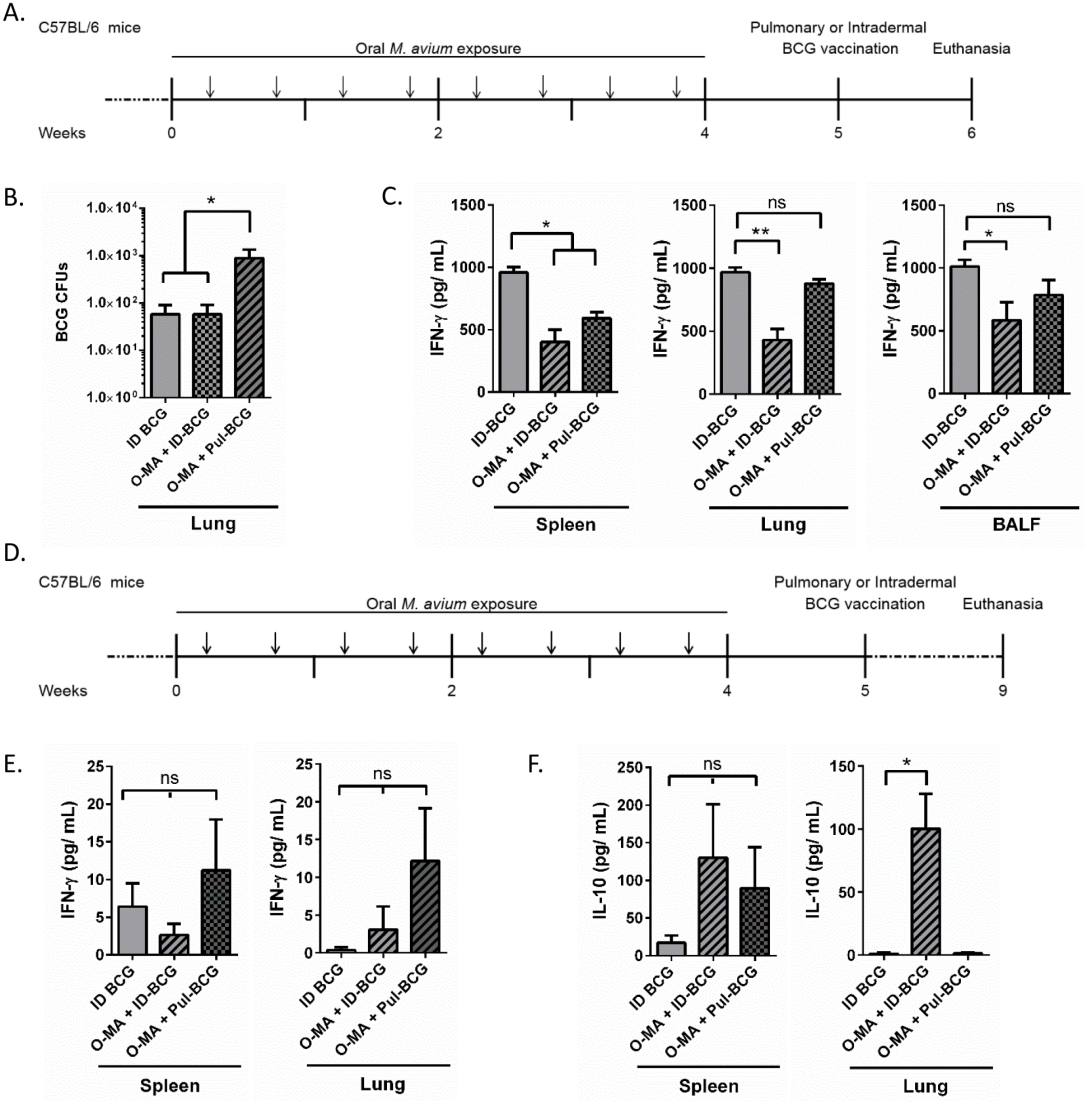
Pulmonary versus intradermal BCG immunization. (A) Experimental design for B & C. Arrows indicate *M*. *avium* exposure, (B) Number of BCG CFU in lungs of different immunization groups one week after immunization, n = 6 over two separate experiments. (C) One week post immunization IFN-γ production by BCG-stimulated T cells from the spleen, lung, and BALF, n = 6 over two separate experiments, (D) Experimental design for E & F. Arrows indicate *M*. *avium* exposure, (E-F) Four weeks post-immunization IFN-γ (E) and IL-10 (F) production by BCG-stimulated T cells from the spleen and lung, n = 4. A one-way ANOVA Kruskal-Wallis with Dunn’s multiple comparison post-test was used to determine statistical significance. *p < .05; **p < .01; data shown with standard error of the mean (SEM). Abbreviations: Intradermal BCG only (ID-BCG), oral *M*. *avium* presensitization with intradermal BCG vaccination (O-MA + ID-BCG), oral *M*. *avium* presensitization with pulmonary BCG vaccination (O-MA + Pul-BCG).

Presence and persistence of antigen in the lung is critical for the generation of long-term tissue-resident immunity and protection against Mtb [20,32–36]. Therefore, we assessed the ability of BCG to survive and migrate to the lung following intradermal versus pulmonary immunization (Pul-BCG), using the previous experimental design (Fig 3A). We observed that one week after immunization, both with and without prior *M*. *avium* exposure, intradermal BCG immunization resulted in minimal BCG CFUs in the lung tissue (Fig 3B). Comparatively, pulmonary BCG immunization (O-MA+Pul-BCG) resulted in a log increase in BCG CFUs in the lung with prior oral *M*. *avium* exposure. These data suggest that intradermal BCG vaccination may result in limited migration of BCG to the lung [6].

In addition to generating tissue resident immunity, various research suggests that pulmonary immunization may be the only way to generate immunity in the airways [26,28,37]. Concurrently, we inferred that systemic tolerance generated by *M*. *avium* would not be present in the airway. To test initial responses to *M*. *avium* exposure we compared IFN-γ production by T cells in response to restimulation with heat-killed BCG in the *M*. *avium*-exposed, BCG-immunized groups (O-MA+ID-BCG, O-MA+Pul-BCG) compared to the intradermal-only BCG group (ID-BCG) (Fig 3C). Compared to those from the ID-BCG group, splenic T cells from the O-MA+ID-BCG and O-MA+Pul-BCG groups produced significantly less IFN-γ. However, in both the lung and airway, only T cells from the O-MA+ID-BCG group showed significantly reduced IFN-γ production compared to the ID-BCG control, whereas O-MA+Pul-BCG had similar IFN- γ levels to that of the ID-BCG group (Fig 3C). These data suggested that O-MA-mediated tolerance is not present in the airways.

We again assessed both IFN-γ and IL-10 levels in these groups at 4 weeks after BCG vaccination to determine whether BCG vaccination had changed initial immune responses to *M*. *avium* by this later time point (Fig 3D). While there was no significant difference in IFN-γ secretion between the two groups, spleens of O-MA+ID-BCG treated mice secreted less IFN-γ than spleens of mice given ID-BCG alone (Fig 3E, spleen). Furthermore in both the spleen and lung, restimulated cells from O-MA+Pul-BCG mice secreted the most IFN-γ (Fig 3). With respect to IL-10 secretion in the spleen, O-MA exposure in both BCG vaccination groups increased secretion levels (Fig 3F, spleen), however this did not reach statistical significance. Conversely, in the lung, IL-10 levels were increased in the O-MA+ID-BCG group but not in lungs from the O-MA+Pul-BCG or ID-BCG treatment mice (Fig 3F, lung).

### Pulmonary BCG vaccination increases correlates of protection in the airway of EM-exposed mice

**Fig 4:**
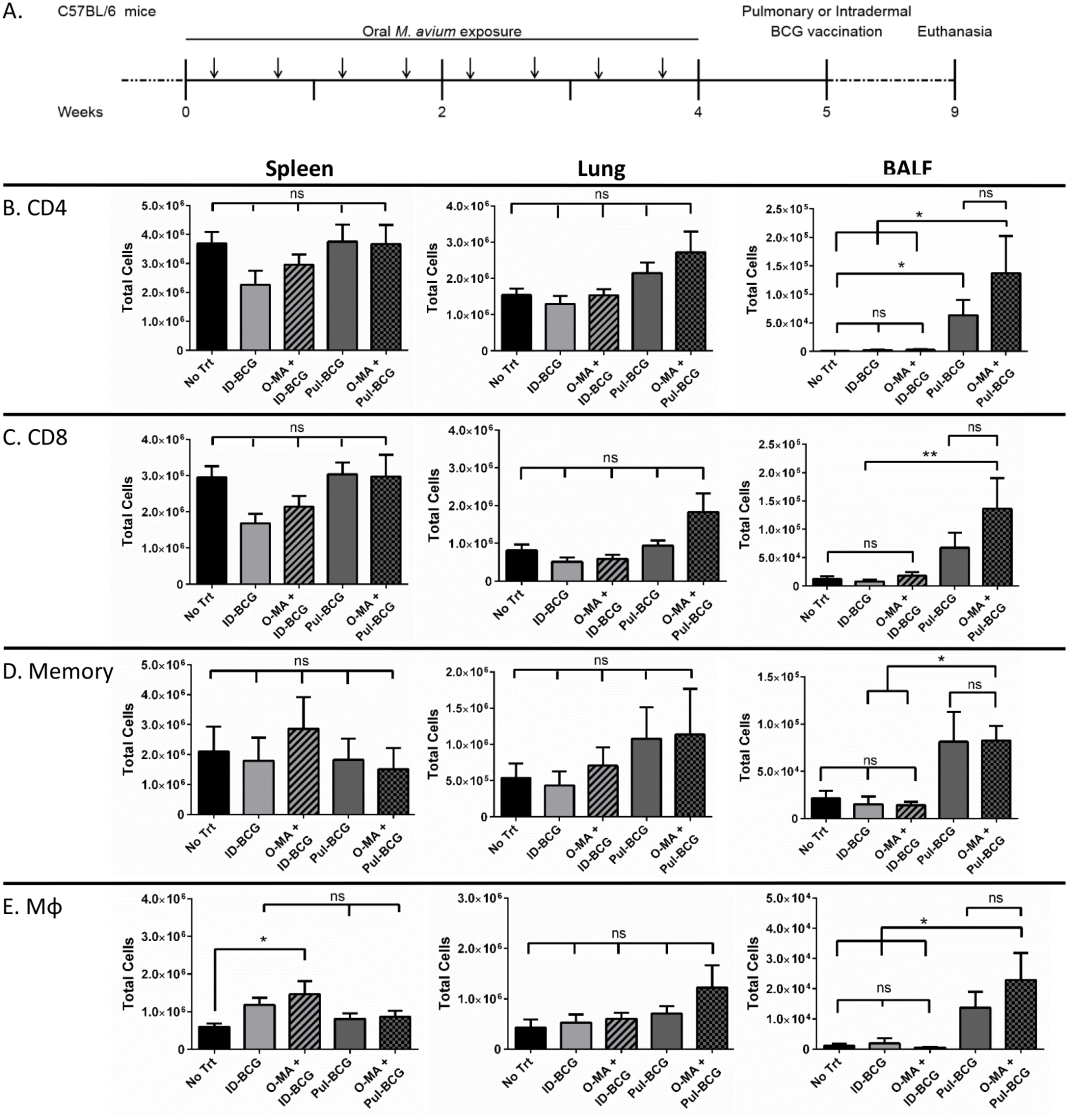
Analysis of cellular correlates of protection in different immunization groups with and without oral *M*. *avium* presensitization. (A) Experimental design. Arrows indicate *M*. *avium* exposure. (B) CD4^+^ cells, (C) CD8^+^ cells, (D) CD44^hi^CD62^lo^ memory cells, (E) CD11b^+^ (and CD11c^+^ for lung and BALF) macrophages present in the spleen, lung, and BALF (airway) compartments, n = 8 over 2 separate experiments. A one-way ANOVA Kruskal-Wallis with Dunn’s multiple comparison post-test was used to determine statistical significance. *p < .05; data shown with standard error of the mean (SEM). Abbreviations: Naïve mice with no presensitization or vaccination (No Trt), intradermal BCG only (ID-BCG), oral *M*. *avium* presensitization with intradermal BCG vaccination (O-MA + ID-BCG), pulmonary BCG only (Pul-BCG), oral *M*. *avium* presensitization with pulmonary BCG vaccination (O-MA + Pul-BCG).

To further evaluate the efficacy of pulmonary BCG vaccination in EM-exposed mice, we measured known immunological correlates of protection against TB, including CD4^+^ T helper cells, CD8^+^ cytotoxic T cells, CD44^hi^CD62L^lo^ effector memory T cells, and macrophages. C57BL/6 mice were chronically exposed to oral *M*. *avium*, rested for 1 week, and then vaccinated with BCG by either the intradermal (O-MA+ID-BCG) or pulmonary (O-MA+Pul-BCG) route (Fig 4A). After 4 weeks, mice were euthanized and bronchioalveolar lavage fluid (BALF), lung, and spleen were harvested to assess the number of inflammatory cells. (Fig 4B–4E).

Lung and spleen homogenates showed minimal differences in immune cell numbers (CD4^+^, CD8^+^, CD44^hi^CD62L^lo^ memory, and macrophages) regardless of immunization route (Fig 4; Spleen & Lung panels). Interestingly, both the lung and spleen tissues showed diminished CD4^+^ and CD8^+^ T cells in the O-MA+ID-BCG group compared to the O-MA+Pul-BCG; however, these differences were not significant. Conversely, the airway (BALF panel) of the pulmonary-vaccinated groups had significantly higher immune cell numbers compared to other groups, and importantly, O-MA exposure did not diminish the Pul-BCG-generated immune response in this compartment (Fig 4; BALF panels). Together with the rescued IFN-γ levels associated with O-MA+Pul-BCG (Fig 3C), these data suggest that pulmonary BCG vaccination generates better correlates of protection in the airway than intradermal BCG in an *M*. *avium*-exposed host.

### Pulmonary BCG vaccination protects EM-exposed mice against Mtb

**Fig 5:**
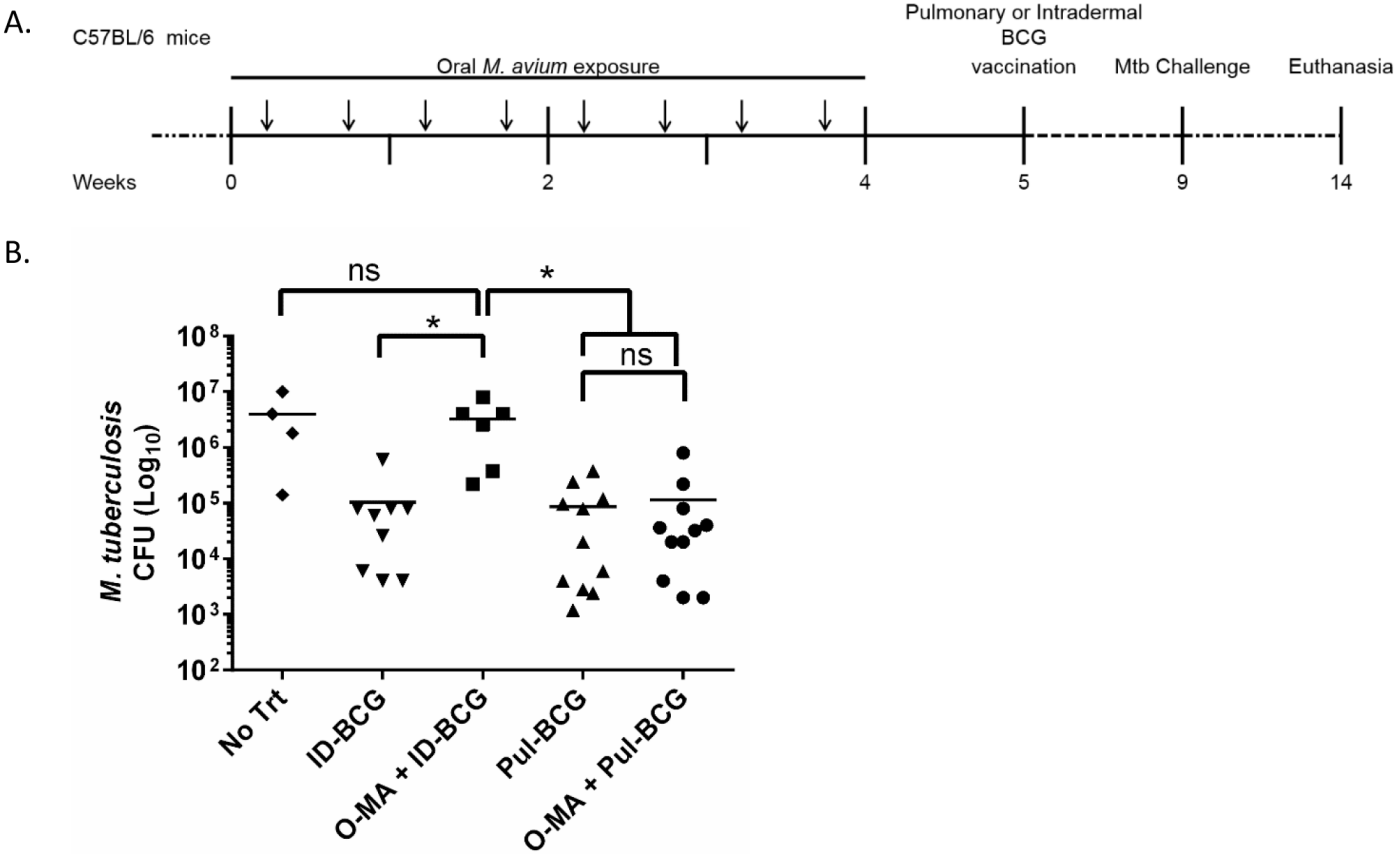
Mtb aerosol challenge studies. (A) Experimental design. Mice immunized with BCG by the intradermal and pulmonary route, with and without presensitization with oral *M*. *avium*, were challenged with Mtb. (B) Number of Mtb CFU in lungs of different immunization groups, n = 4–12 per group (controls started with n = 8 over two independent experiments and treatment animals with an n = 12, over four independent experiments), where only mice which survived and had successful infection were included (mice lost post-infection No Trt (2), OMA+ID-BCG (2)). A one-way ANOVA Kruskal-Wallis with Dunn’s multiple comparison post-test was used to determine statistical significance. *p < .05; data shown with standard error of the mean (SEM). Abbreviations: Naïve mice with no presensitization or vaccination (No Trt), intradermal BCG only (ID-BCG), oral *M*. *avium* presensitization with intradermal BCG vaccination (O-MA + ID-BCG), pulmonary BCG only (Pul-BCG), oral *M*. *avium* presensitization with pulmonary BCG vaccination (O-MA + Pul-BCG).

The ultimate measure of any vaccine’s efficacy is pathogen challenge. Therefore, to test whether pulmonary BCG could protect EM-exposed mice against Mtb challenge, C57BL/6 mice were exposed chronically to oral *M*. *avium* as performed before (Fig 5A). Mice were rested for 1 week and then vaccinated with BCG by either the intradermal or pulmonary route. After 4 weeks, mice were challenged with aerosolized Mtb. Mice were rested for 5 weeks before euthanasia, and tissues were assessed for Mtb burden by plating for CFUs.

An *M*. *avium* only group was not included in the challenge study, as it has been shown previously that *M*. *avium*, both by the oral and parenteral route, does not offer protection against aerosol Mtb challenge [6–8]. Mouse spleens showed no detectable Mtb CFUs at 5 weeks post-infection by plating. The lungs of unvaccinated Mtb-challenged (No Trt) mouse controls showed marked pulmonary infection, with greater than 10^6^ Mtb CFUs present in the lung 5 weeks after aerosol infection (Fig 5B), whereas ID-BCG and Pul-BCG immunization significantly diminished TB burden. In agreement with previous studies [6], O-MA+ID-BCG mice showed Mtb bacterial numbers in the lung equal to that of the unvaccinated controls, confirming that *M*. *avium* exposure renders intradermal BCG ineffective [3,6–9]. Importantly, Pul-BCG and O-MA+Pul-BCG mice showed similar and significant Mtb clearance from the lung, compared to No Trt and O-MA+ID-BCG. This confirms that pulmonary BCG vaccination can circumvent the immune suppression caused by previous chronic oral *M*. *avium* exposure (Fig 5B).

Changes in lung tissue and mouse weight five weeks after Mtb infection were used to assess morbidity. There was no difference in total lung weight among groups (S3B Fig); however, O-MA+ID-BCG mice showed decreased weight gain after Mtb infection compared to the immunization control mice (ID-BCG), suggesting increased morbidity following prior *M*. *avium* exposure (S3C Fig).

**Fig 6:**
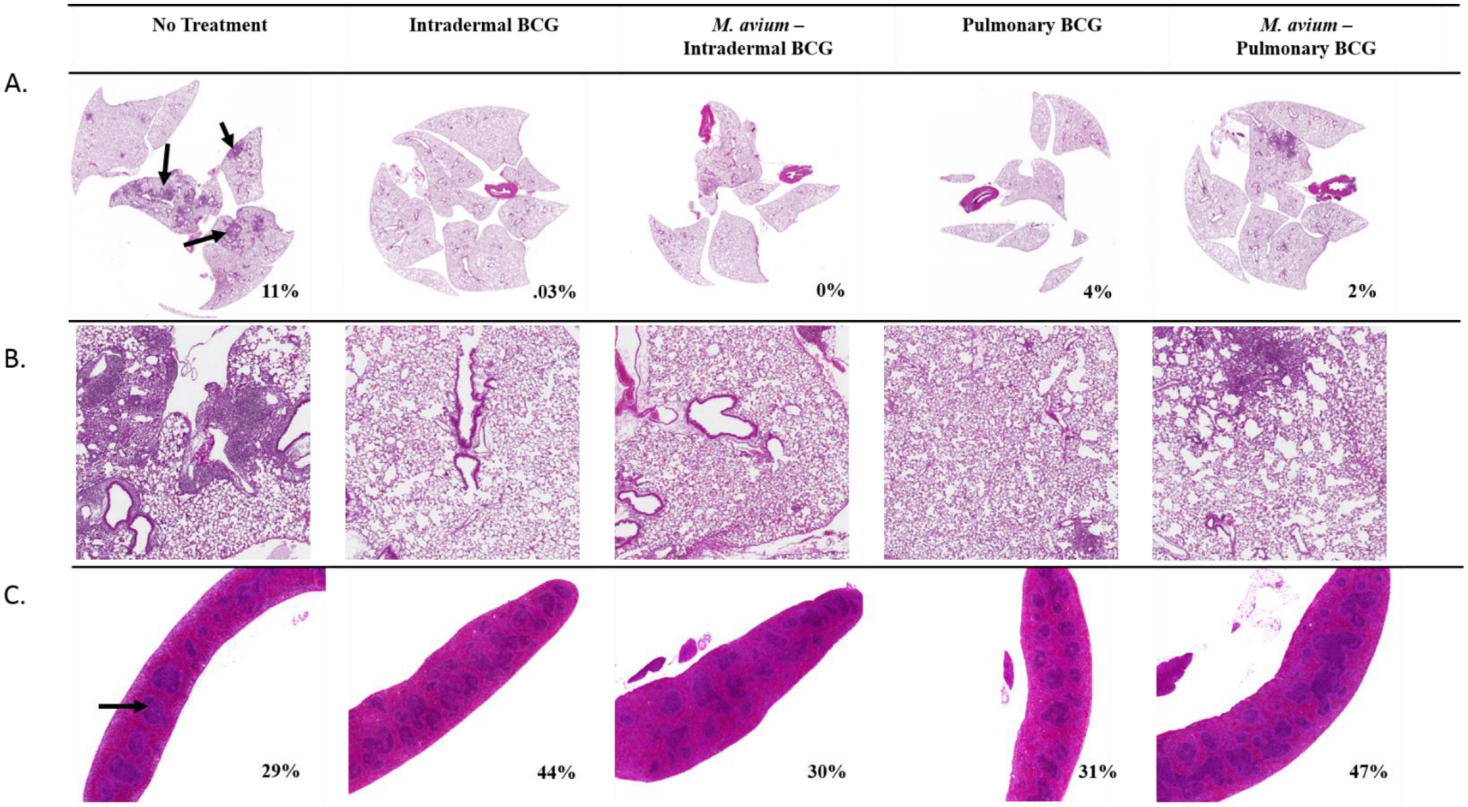
Histopathological analysis of lung and spleen tissue after Mtb aerosol challenge in different immunization groups, with and without oral *M*. *avium* presensitization. (A) H&E-stained lung sections were assessed for granulomatous inflammation, arrows indicate foci of granulomatous inflammation. (B) Higher magnification (5X) of lung sections. (C) Spleens were assessed for lymphoid hyperplasia, the arrow indicates an area of white pulp used to assess lymphoid hyperplasia. n = 2 mice per group, data shown is from one experiment and representative of three independent experiments.

To assess differences in tissue inflammation between *M*. *avium*-exposed and BCG-immunized mice following Mtb challenge, lungs and spleens were excised and stained with hematoxylin and eosin (Fig 6). Lungs of untreated animals (No Trt) showed considerable pathology, with granulomatous lesions occupying an average of 11% of the area of lung sections (Fig 6A & 6B). Pul-BCG and ID-BCG groups showed minimal inflammation, with inflammatory infiltrates making up 4% and 0.03% of the lung area, respectively. O-MA+ID-BCG and O-MA+Pul-BCG groups also had minimal inflammation; with lesions occupying less than 2% of the area of lung sections for each group. Although these differences are slight, they may suggest that Pul-BCG immunization causes more inflammation in the lung than ID-BCG immunization. Lymphoid hyperplasia in the spleen, assessed by quantifying the relative area of the white pulp in each spleen section, was used as an indirect measure of the systemic immune response to Mtb challenge (Fig 6B). In No Trt mice, the white pulp occupied an average of 29% of the spleen area. Both BCG-vaccinated groups had higher percentages of white pulp, with 44% for ID-BCG and 31% for Pul-BCG groups. O-MA+ID-BCG animals had a white pulp area of 30%, a reduction of 14% compared to the ID-BCG only group. The O-MA+Pul-BCG group had a white pulp area similar to that of ID-BCG, with a total white pulp area of 47%. Taken together, these findings suggest that the systemic immune response after ID-BCG vaccination is inhibited by *M*. *avium* presensitization, whereas the systemic immune response to pulmonary BCG vaccination with prior *M*. *avium* exposure was similar to intradermal vaccination in naïve animals.

## Discussion

The BCG vaccine is given by the intradermal route in over 150 countries worldwide [38]. However the vaccine’s efficacy is impaired in areas where EM are endemic [4,5]. In these areas of the world, EM is ubiquitous in soil, air, and water [4,11,12].

Prior EM exposure has been shown to be detrimental to BCG protective efficacy in mice [3,6,7,9,39]. Previously, two mechanisms have been proposed for the failure of BCG due to EM exposure, the blocking and the masking hypotheses [40], which propose that BCG replication is blocked or BCG immunity is masked by EM, respectively. Here we show evidence for an alternate mechanistic hypothesis, BCG vaccine interference via oral tolerance, which may encompass both the blocking and masking hypotheses [40]. Importantly, we presensitized mice via the oral route to mimic human ingestion of EM (without antibiotic clearance) similar to Flaherty and colleagues [7], whereas other early studies used parenteral exposure with antibiotic clearance of the EM before vaccination [6,8,41]. To our knowledge, this is the first study to demonstrate oral tolerance as a mechanism of BCG vaccine failure.

We used a mouse model to investigate the mechanism of EM interference by exposing mice to *M*. *avium* (a common EM) via the oral route [3,9]. Mice were then vaccinated intradermally with BCG. The immune response of mice, based on IFN-γ production by T cells following *ex vivo* BCG restimulation, was significantly decreased in mice exposed to *M*. *avium* prior to intradermal vaccination (Fig 1C). This corroborates previous findings that *M*. *avium* exposure impairs the immune response to BCG [3,6,7,9,39]. Interestingly, the masking hypothesis states that a naïve host produces a lower initial pro-inflammatory response to EM presensitization. It should be noted that this immune response can be cross-reactive against Mtb, but is not as strong as the immunity generated by BCG vaccination in a naïve host [41]. After EM presensitization, individuals vaccinated with BCG cannot produce a pro-inflammatory response to BCG that ever surpasses the initial pro-inflammatory response to EM; thus, the immune response to EM masks the subsequent immune response generated against BCG [40,41]. Although our data, as well as those of others, initially appeared to support the masking hypothesis, we further explored the mechanism of BCG vaccine interference [41,42].

We examined the cross-reactivity of T lymphocytes to *M*. *avium* and BCG by taking T cells from ID-BCG or O-MA exposed mice and restimulating with either antigen *ex vivo*. We observed identical IFN-γ responses in tissue-specific T lymphocyte populations from both exposure groups (Fig 1B). This suggests that the immune cells cannot distinguish between the live BCG vaccine and *M*. *avium*. Therefore, the EM immune response does not “mask” the BCG-specific response, but rather this immunosuppression is a consequence of cross-reactivity between the two mycobacterial species, wherein the host immune system cannot distinguish between *M*. *avium* and *M*. *bovis* BCG.

Previously, both Flaherty et al. and Poyntz et al., showed reduced BCG efficacy associated with oral *M*. *avium* exposure [3,7]. To determine if the immunosuppressive effect of *M*. *avium* depended on the route of *M*. *avium* exposure, mice were administered *M*. *avium* chronically by either the oral or by the intradermal route. Mice exposed to *M*. *avium* by the oral route, but not by the intradermal route, had elevated Treg cells in the spleen (Fig 2B) and increased IL-10 secretion by both splenic and lung T cells in response to BCG restimulation at multiple timepoints after vaccination (Figs 1D, 3F & S1). Importantly, Treg cells and IL-10 are both mediators of oral tolerance [22,24]. This observation is also supported by both data generated in mouse models of EM exposure [3,43,44], and some epidemiological data examining patients with EM infections [45]. These individuals present with increased T regulatory numbers, along with the associated cytokines [45]. This, coupled with *M*. *avium* colonization of the GI tract in mice exposed orally (Fig 2D), suggests that the immunosuppressive effects of EM are closely linked to the oral route of exposure.

**Fig 7:**
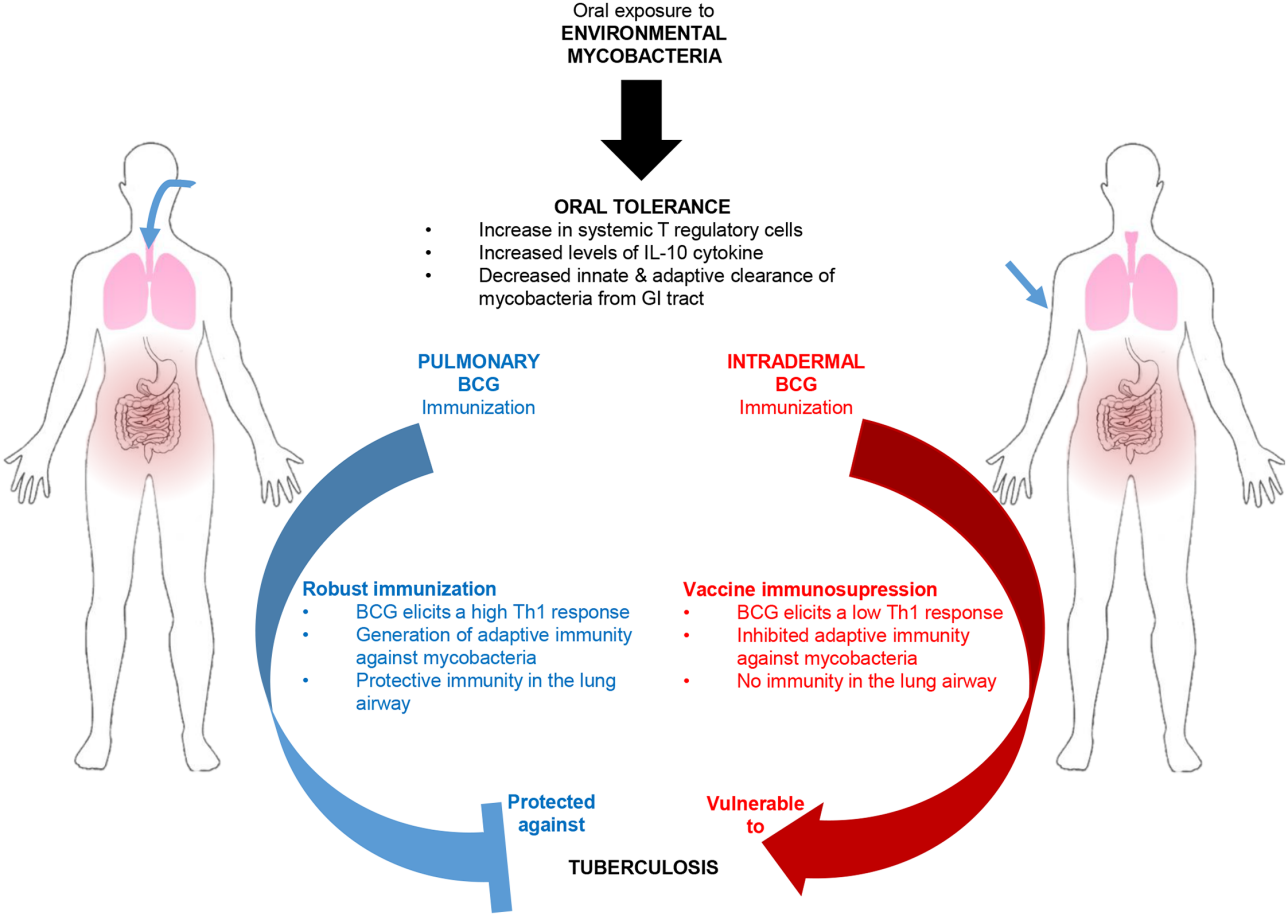
Pulmonary BCG immunization is effective at protecting against Mtb in EM exposed hosts. Chronic oral exposure to EM results in systemic tolerance against mycobacteria. This tolerance generates poor immunity in a subsequent intradermal BCG vaccination scenario, and could lead to variable vaccine efficacy. However, because systemic tolerance is not present in the airways of hosts, pulmonary immunization generates robust immunity and is protective against Mtb challenge.

Another mechanism suggested to explain BCG vaccine interference by EM is described by the blocking hypothesis [6]. This hypothesis suggests that BCG is eliminated in hosts after vaccination due to prior EM immunity. This is detrimental because BCG is required to migrate to the lung and persist long enough to generate BCG-specific immunity [35,36]. However, early animal experiments supporting this hypothesis did not utilize oral EM administration, the most common route of human exposure, thus oral tolerance was not observed [6]. Our study shows that the immunosuppression against BCG vaccination associated with *M*. *avium* is linked to the oral route of exposure. Only oral *M*. *avium* exposure increased splenic and lung Treg cells (Fig 2B). Furthermore, with respect to the blocking hypothesis, we show that BCG migrates minimally to the lung after intradermal immunization, regardless of prior EM exposure (Fig 3A). Previous literature suggests that this may be due to a delay in migration associated with intradermal immunization [46], however the result is that a robust local immune response to BCG is not generated in the lung. Contrarily, mice vaccinated by the pulmonary route had more BCG in the lung one week after immunization (6 weeks after initial *M*. *avium* exposure) and thus produced a Th1 immune response. These data support our hypothesis that EM immunosuppression is an oral tolerance mechanism, and that the route of EM exposure and BCG immunization are both important (Fig 7).

Our next step was to investigate whether EM-driven immunosuppression could be overcome by delivering the BCG vaccine by the pulmonary route. Horvath and colleagues demonstrated that the pulmonary airway is an isolated immune compartment which can only be effectively immunized against TB via the pulmonary route [28]. According to these authors, parenteral vaccination is sufficient to generate immune cells in the systemic circulation and in the lung parenchyma; however, only intranasal immunization generates an adaptive immune response in the lung airway compartment. We hypothesized that the airway would be immunologically naïve to EM-generated systemic tolerance induced via a gut-mucosa-specific mechanism. As expected, mice vaccinated with BCG by the pulmonary route after oral *M*. *avium* exposure had significantly higher Th1 responses in the airway, as demonstrated by the large number of T-lymphocytes (both helper and cytotoxic), effector memory cells, and macrophages present in BALF (Fig 4; BALF panel), compared to *M*. *avium*-exposed mice subsequently vaccinated intradermally with BCG. Most importantly, we showed that oral EM exposure impairs the efficacy of intradermal, but not pulmonary, BCG vaccination against subsequent aerosol Mtb challenge (Fig 5B). These results show that pulmonary BCG immunization is effective at protecting against Mtb, even in EM-exposed individuals.

To determine the broader implications of these studies, future experiments should test the ability of multiple strains of orally administered EM to generate tolerance. It has been shown that EM exposure in humans is variable on both the species and dosing level, with *M*. *avium*, *M*. *fortuitum*, *M*. *parafortuitum*, *M diernhoferi*, and *M*. *phlei* all being present in variable amounts in the human sputum samples [4]. Likewise, similar studies in small animal models have previously shown that different species and strains of EM cause variations in the immunosuppressive effects seen against intradermal BCG vaccination [3,6,7,9]. Also, studies done previously show little to no protection provided by parenteral *M*. *avium* alone against Mtb challenge [6,41]. Similarly, Flaherty and colleagues showed that oral *M*. *avium* alone resulted in Mtb burden similar to that seen in naïve control mice [7]. Therefore, it is important to investigate how tolerance against these different EM might affect Mtb infection as well as BCG immunization.

Furthermore, many of the details of this tolerance mechanism still remain unknown. Does contiguous oral EM exposure before and after BCG vaccination still result in tolerance? We and others have shown how oral and parenteral EM presensitization prior to vaccination affects BCG protective efficacy [6,9,39]. Likewise, others have examined the effects of oral and parenteral EM sensitization after BCG vaccination [3,7]. It is more likely that EM exposure is consistently present throughout the life of these individuals. Most countries vaccinate with BCG in the first few years of life [38]. However many would argue that this is the most critical time period for GI flora generation [47,48]. Infants would be extremely vulnerable to EM colonization, and may even become sensitized to the EM on their mother’s skin and in her breast milk [49]. In this case, one might expect that after vaccination any BCG-specific immunity generated would predictably wane as a result of continuous tolerance against EM. Thus, the question of how and when exposure happens remains critical to understanding how BCG vaccine immunosuppression takes place in humans.

Lastly, it is important to examine how pulmonary EM exposure affects immunity in the airway. The airways tend to be a reactive tissue and rarely tolerogenic. It may be that EM exposure is beneficial in the airway providing a boost to BCG established immunity. Research published in 1984 by Orme and Collins showed that a single pulmonary exposure of *M*. *avium* and other EM conferred significant TB resistance [50]. While this seems to suggest that pulmonary EM exposure was not immunosuppressive in nature, this study should be repeated with a chronic exposure experimental design to ensure that no tolerance is generated. Chronic EM exposure by the pulmonary route could prove technically challenging to study due to mucociliary clearance from the airways and subsequent ingestion of the EM. Regardless of the difficulties, these questions must be answered to completely understand the mechanisms behind the failures and successes of prospective TB vaccines currently in clinical trials.

This work helps us to understand the limitations of the current BCG vaccine and the potential for failure of any new vaccine administered by the parenteral route [51]. If oral tolerance interferes with the intradermal BCG vaccine in humans, then it has major implications for vaccines against TB and other infectious diseases. Since EM are ubiquitous soil and water residents, little can be done to avoid human exposure. Moreover, the effects of oral tolerance are not tissue-specific. Tolerance is generated in the GI tract, and tolerogenic T cells migrate throughout the body via the circulation [22,24]. This may result in the failure of any new intradermal TB vaccine that uses mycobacterial antigens. We have demonstrated that pulmonary immunization will circumvent EM-generated tolerance and provide protective immunity against Mtb. Importantly, this immunity is evident in the airways, the tissue compartment where the host first encounters this important human pathogen.

## Methods

### Animals

Female, pathogen-free, 6–12 week old C57BL/6 mice were purchased from Jackson Laboratory (Bar Harbor, ME). Animals were kept in cages with laminar flow safety enclosures and housed by groups. Mice were acclimatized for at least one week in a climate-controlled room on a 12-hour light-dark cycle and were fed ad libitum prior to starting the experiments. All experiments were approved by the UNM HSC IACUC (Protocol #12-100817-HSC) and conformed to the *Guide for the Care and Use of Laboratory Animals* [52].

### Mycobacteria

*Mycobacterium avium* (ATCC 700898), *Mycobacterium bovis BCG Pasteur* (ATCC 35734), and *Mycobacterium tuberculosis* strain H37Rv (ATCC 27294) were grown in Middlebrook 7H9 broth (Fluka analytical, Buchs, Switzerland) supplemented with 0.05% glycerol (Fisher Scientific, Waltham, MA) and 10% albumin-dextrose-catalase (BD diagnostic Systems, Franklin Lakes, NJ) at 37°C to mid-log phase of growth. *M*. *avium* was chosen as the experimental EM species because of its use in earlier literature and because of its ability to cause persistent infections in humans and mice [3,6,7]. In preparation for oral gavage, intradermal exposure or vaccination, and aerosol administration- *M*. *avium*, BCG, and Mtb were pelleted at 1800*g* and resuspended in saline. Harvested tissue homogenates were plated on Middlebrook 7H10 agar (Fluka analytical, Buchs, Switzerland) supplemented with 0.05% glycerol (Fisher Scientific) and 10% oleic acid-albumin-dextrose-catalase broth for 14–28 days at 37°C. Mycobacterial strains were differentiated by supplementing agar with clarithromycin (Sigma Aldrich, St. Louis, MO, 2μg/mL) and/or 2-thiophenecarboxylic acid hydrazide (TCH) (Sigma Aldrich, 2μg/mL) to exclude *M*. *avium* or BCG, respectively. *M*. *avium* in feces was collected (by group) and 75mg was suspended in 1mL before plating for analysis on plates containing 2-thiophenecarboxylic acid hydrazide to exclude BCG. Tissues for Mtb enumeration were plated with both clarithromyocin and TCH to block both BCG and *M*. *avium* growth.

### Sensitization with environmental mycobacteria

Mice were exposed to a 50μL dose of *M*. *avium* orally (2 x 10^6^ CFU) by gavaging with an 18-gauge blunted needle (O-MA) (Fine Science Tools, Foster City, CA) or by intradermal injection (ID-MA) on the flank (2 x 10^6^ CFU). *M*. *avium* exposure was repeated two times a week for four weeks (total 8 exposures). Dosing CFUs were taken from earlier literature, and timelines were created to mimic chronic oral exposure to EM by humans [6,7,39].

### Vaccination

The dosing of BCG followed earlier literature [6] and the intradermal route was chosen as this is the current route of immunization in humans. Mice were vaccinated one week after the last presensitization with *M*. *avium*. A single 50μL dose of 5x10^7^ CFU BCG was given by the intradermal route (ID-BCG) on the flank or by endotracheal aerosolized (Pul-BCG) delivery using a MicroSprayer Aerosolizer (Penn Century, Wyndmoor, PA) while mice were anesthetized with constant 3% isoflurane. Mice were sacrificed either one week (six weeks after the first *M*. *avium* exposure) or five weeks (nine weeks after the first *M*. *avium* exposure) after BCG immunization.

### Aerosol TB infection

Mice were infected with a low-dose aerosol Mtb (200 CFU) four weeks after vaccination via endotracheal aerosolization using the Penn Century MicroSprayer Aerosolizer while anesthetized with ketamine and xylazine (10+100mg/kg). Mice were sacrificed five weeks after aerosol infection and lungs and spleens were plated for Mtb growth. Mice which died or that showed no infection (by plating) were not included.

### Tissue preparation

Mice were sacrificed one week after vaccination (oral tolerance experiments) or five weeks after vaccination (correlates of protection experiments). Tissue samples were collected and prepared as described below.

### Splenic lymphocytes

Isolated spleens were placed in a tube containing ice cold Dulbecco’s Phosphate Buffered Saline (DPBS) (Gibco, UK) supplemented with 2.5% fetal bovine serum (FBS) (Life Technologies, Carlsbad, CA). Spleens were dissociated using mouse spleen dissociation protocol 1 on a gentleMACS tissue dissociator (Miltenyi Biotec Ltd., Bergisch Gladbach, Germany). Dissociated spleens were pushed through 40μm mesh cell strainers (Fisher Scientific, Fisher Scientific) and pelleted at 300g. Pellets were resuspended in 5mL of MACS Red Blood Cell Lysis Solution (1X) (Miltenyi Biotec Ltd.) and incubated for 10 minutes at room temperature. Splenic cells were again pelleted and resuspended in 5mL of Dulbecco’s Modified Eagle Medium (DMEM) (Gibco).

### Lung tissue

Lung tissue was isolated, and placed in a tube containing ice cold dissociation buffer consisting of DPBS supplemented with 2.5% FBS, 40U/mL DNAse 1 (Sigma Aldrich), and 150U/mL Collagenase 1 (Sigma Aldrich). Lungs were partially dissociated using the mouse lung dissociator program 1 on a gentleMACS tissue dissociator. Lung tissue was then incubated in the dissociation buffer for 1 hour at 37°C on a shaker. After incubation lung tissue was further dissociated on the gentleMACS tissue dissociator using mouse lung dissociation program 2. Dissociated lungs were then pushed through 40μm mesh cell strainer and the cells were pelleted at 300g. Pellets were resuspended in 5mL of MACS Red Blood Cell Lysis Solution (1X) and incubated for 10 minutes at room temperature. Lung cells were again pelleted and resuspended in 5mL of DMEM.

### Immunophenotyping via flow cytometry

1x10^6^ cells per tissue preparation were stained for flow cytometry according to the antibody manufacturer’s instructions (eBiosciences, San Diego, CA). Briefly, anti-CD4 PerCP-Cy5.5, CD8 APC-eFluor 780, CD11b PE-Cyanine7, CD11c eFluor 450, CD3 APC, CD44 FITC, CD62L eFluor 605NC, and anti-FoxP3 PE (after fixation and permeabilization) antibodies were incubated with cells for 30 minutes in the dark at 4°C and cells were then fixed with 4% paraformaldehyde. Cells were resuspended in FACS buffer and samples were analyzed on the LSRFortessa Flow Cytometer (Becton Dickinson, Franklin Lakes, NJ). Lymphocytes were gated based on their forward- and side-scatter profiles and the presence of CD3, and then sub-gated on CD4 (with further sub-gating on FoxP3 for CD4^+^FoxP3^+^ cells), CD8, and CD44^hi^CD62L^lo^. Macrophages were gated on their forward- and side-scatter profiles and the presence of CD11b, with sub-gating in lung homogenates on CD11c for alveolar macrophages.

### T-cell restimulation and ELISA

Lymphocyte preparations were counted and 10^6^ cells plated per well in a 24-well round bottomed plate with DMEM media supplemented with 10% FBS. Heat killed BCG was added to the cells at a multiplicity of infection (MOI) of 5. Cells were incubated at 37°C for 48 hours in 5% CO_2_. Cell supernatants were collected and assessed for cytokines using either Milliplex bead-based cytokine kits (EMD Millipore, Billerica, MA) or using the Ready-Set-Go ELISA (eBiosciences). ELISAs were read on a Tecan Microplate reader (Mannedorf, Switzerland) and Milliplex assays were measured on a Bio-Rad Bio-Plex 200 system (Bio-rad, Hercules, CA).

### Histopathology

Mouse lungs and spleens were removed en bloc. Lungs were inflated with 10% paraformaldehyde and both lung and spleen were submerged in 10% paraformaldehyde for one week. Lungs and spleen were embedded in paraffin blocks, sectioned at 4–5μm and stained with hematoxylin and eosin (H&E). Histopathology was performed by a board-certified veterinary pathologist (DFK). H&E-stained slides were digitized for morphometric analysis using an Aperio CS2 slide scanner (Leica, Buffalo Grove, IL) and analyzed using associated morphometry algorithms. Granulomatous inflammation in the lung was determined by manually outlining all foci of inflammation and determining the total area of inflammation as a percentage of the total area of the lung. The area of white pulp in a longitudinal spleen section was obtained by manually outlining all lymphoid aggregates and determining the total area of the white pulp as a percentage of the total area of the spleen.

### Statistical methods

All statistical analysis was performed using Graphpad Prism statistical software (GraphPad Software, San Diego, CA). The Mann Whitney test for non-parametric data of two groups and a Holm-Sidak test was used for multiple t-tests. A one-way ANOVA Kruskal-Wallis with Dunn’s multiple comparison post-test was used for multiple comparisons of non-parametric data. Statistical significance was reported as *, P < .05; **, P < .01; ***, P < .001; ****, P < .0001.

## Supporting Information

S1 Fig Analysis of the cytokines IFN-γ and IL-10 secreted by spleen and lung cells after oral or intradermal presensitization to *M*. *avium*. (A) Experimental design. Arrows indicate *M*. *avium* exposure. (B & C) Comparison of splenic and lung cells restimulated with BCG from mice presensitized with oral versus intradermal *M*. *avium* measuring presence of IFN-γ (B) or IL-10 (C), n = 4. A Mann-Whitney U test was used for comparison of two data sets, *p < .05; data shown with standard error of the mean (SEM). Abbreviations: Intradermal *M*. *avium* only (ID-MA), oral *M*. *avium* only (O-MA).
(TIF)

S2 Fig *M*. *avium* CFUs in lung and spleen after different routes of presensitization. (A) Experimental design. Arrows indicate *M*. *avium* exposure. (B) *M*. *avium* in the lung and spleen of mice presensitized by either the oral or intradermal route, n = 8. A Multiple t-tests (Holm-Sidak) were used for comparison of two data sets, data shown with standard error of the mean (SEM). Abbreviations: Intradermal *M*. *avium* only (ID-MA), oral *M*. *avium* only (O-MA).
(TIF)

S3 Fig Lung tissue and animal weights after infection. (A) Experimental design. Arrows indicate *M*. *avium* exposure. (B) Total lung weight at sacrifice; n = 8. (C) Weight gain after infection; n = 8. A one-way ANOVA Kruskal-Wallis with Dunn’s multiple comparison post-test was used to determine statistical significance. *p < .05; data shown with standard error of the mean (SEM). Abbreviations: Intradermal BCG only (ID-BCG), oral *M*. *avium* presensitization with intradermal BCG vaccination (O-MA + ID-BCG), pulmonary BCG only (Pul-BCG), oral *M*. *avium* presensitization with pulmonary BCG vaccination (O-MA + Pul-BCG).
(TIF)
