# Supplementary figures and images for: Oral Tolerance to Environmental Mycobacteria Interferes with Intradermal, but Not Pulmonary, Immunization against Tuberculosis
Source: PLoS Pathog. 2016 May 6;12(5):e1005614. doi: 10.1371/journal.ppat.1005614 (PMC4859477; doi:10.1371/journal.ppat.1005614)

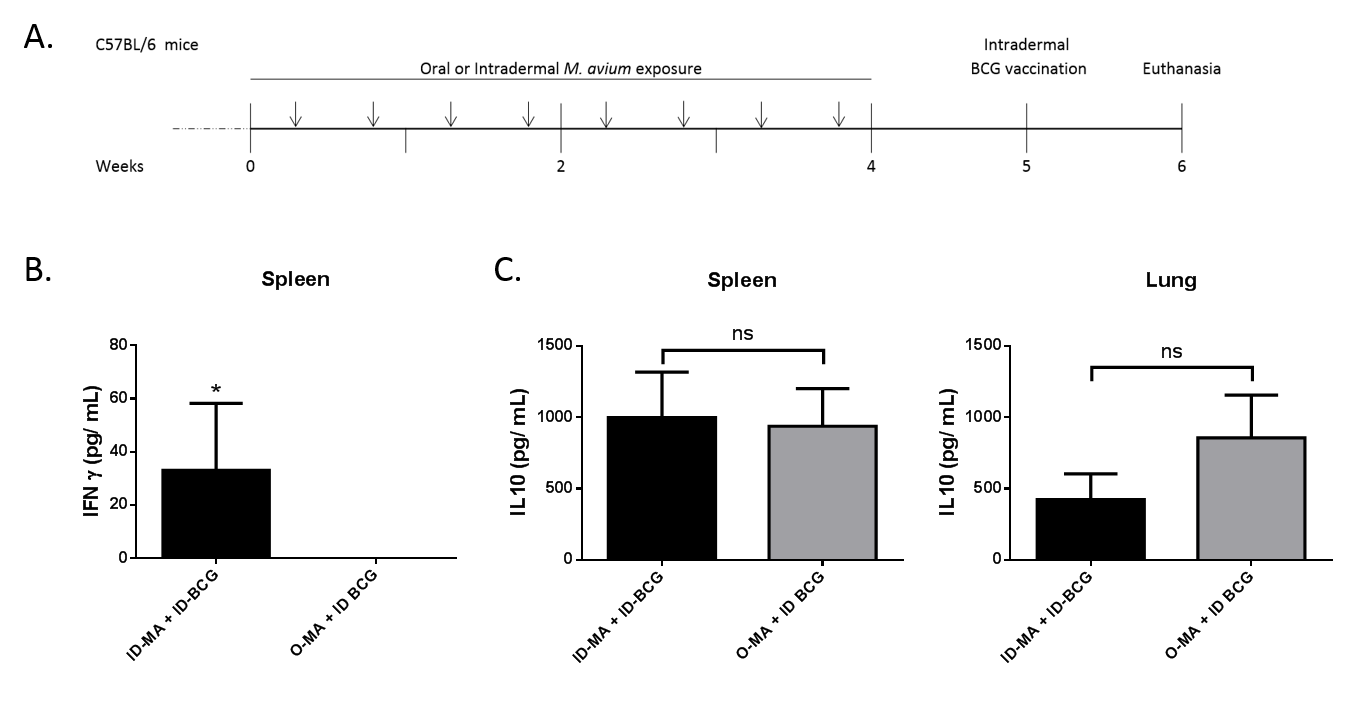

Supplement: S1 Fig — (A) Experimental design. Arrows indicate M. avium exposure. (B & C) Comparison of splenic and lung cells restimulated with BCG from mice presensitized with oral versus intradermal M. avium measuring presence of IFN-γ (B) or IL-10 (C), n = 4. A Mann-Whitney U test was used for comparison of two data sets, *p < .05; data shown with standard error of the mean (SEM). Abbreviations: Intradermal M. avium only (ID-MA), oral M. avium only (O-MA). (TIF) [file ppat.1005614.s001.tif]

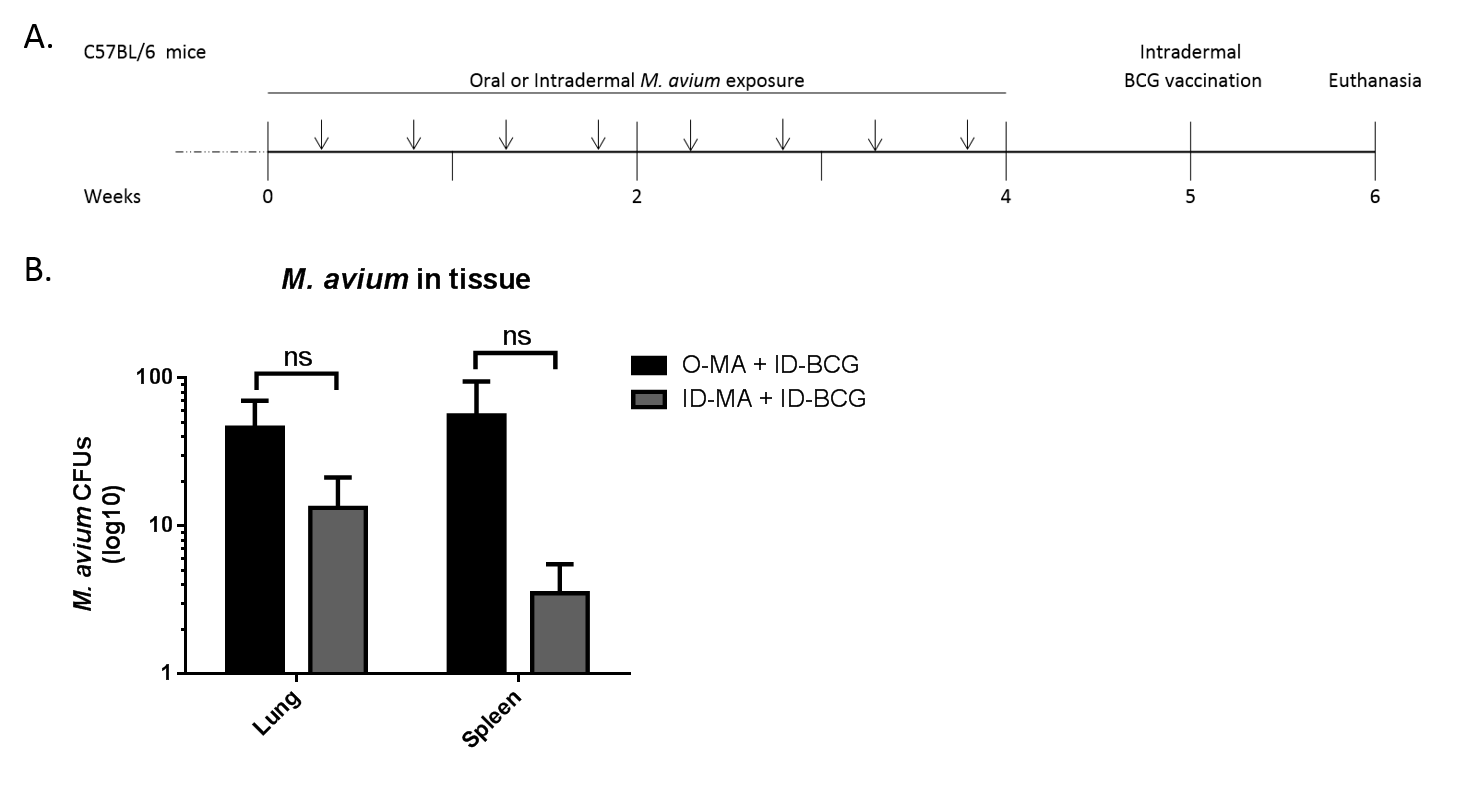

Supplement: S2 Fig — (A) Experimental design. Arrows indicate M. avium exposure. (B) M. avium in the lung and spleen of mice presensitized by either the oral or intradermal route, n = 8. A Multiple t-tests (Holm-Sidak) were used for comparison of two data sets, data shown with standard error of the mean (SEM). Abbreviations: Intradermal M. avium only (ID-MA), oral M. avium only (O-MA). (TIF) [file ppat.1005614.s002.tif]

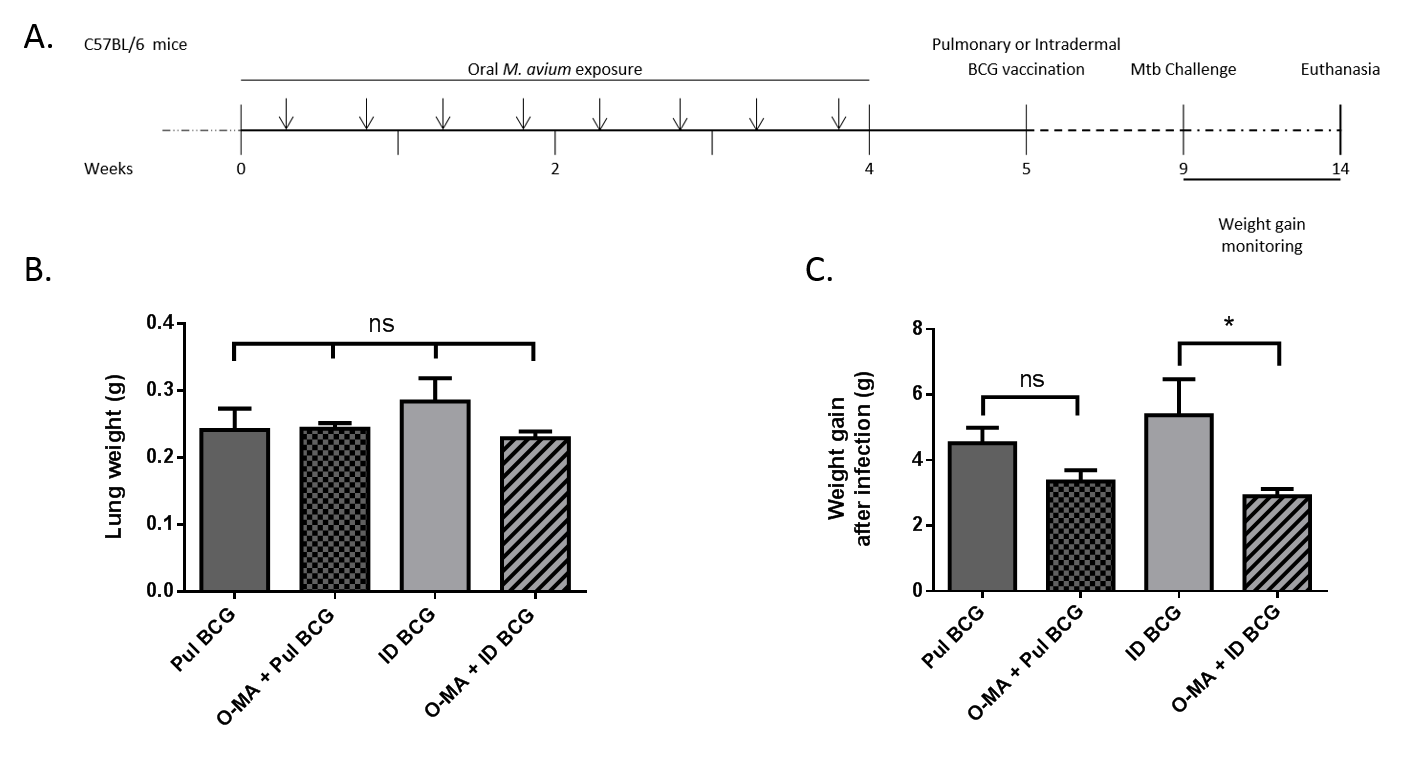

Supplement: S3 Fig — (A) Experimental design. Arrows indicate M. avium exposure. (B) Total lung weight at sacrifice; n = 8. (C) Weight gain after infection; n = 8. A one-way ANOVA Kruskal-Wallis with Dunn’s multiple comparison post-test was used to determine statistical significance. *p < .05; data shown with standard error of the mean (SEM). Abbreviations: Intradermal BCG only (ID-BCG), oral M. avium presensitization with intradermal BCG vaccination (O-MA + ID-BCG), pulmonary BCG only (Pul-BCG), oral M. avium presensitization with pulmonary BCG vaccination (O-MA + Pul-BCG). (TIF) [file ppat.1005614.s003.tif]
